# “*That Guy, Is He Really Sick at All?*” An Analysis of How Veterans with PTSD Experience Nature-Based Therapy

**DOI:** 10.3390/healthcare6020064

**Published:** 2018-06-14

**Authors:** Dorthe Varning Poulsen, Ulrika K. Stigsdotter, Annette Sofie Davidsen

**Affiliations:** 1 Department of Geosciences and Natural Resource Management, University of Copenhagen, Rolighedsvej 23, 1958 Frederiksberg C, Denmark; uks@ign.ku.dk; 2 The Research Unit for General Practice, University of Copenhagen, Øster Farimagsgade 5, 1014 København K, Denmark; adavid@sund.ku.dk

**Keywords:** veterans, post-traumatic stress disorder, nature-based therapy, Interpretative Phenomenological Analysis, qualitative study

## Abstract

Serving in the military leads to mental diseases, such as post-traumatic stress disorder (PTSD), for a percentage of soldiers globally. The number of veterans with PTSD is increasing and, although medication and psychological treatments are offered, treatment results could be improved. Historically, different forms of nature-based therapy have been used for this target group. However, in spite of anecdotally good results, studies measuring the effect of this form of therapy are still lacking. The aim of this study is to explore how veterans with PTSD manage their everyday lives during and after a ten-week nature-based intervention in a therapy garden. Methods: Eight veterans participated in qualitative interviews, which were conducted during a one-year period and were analyzed using interpretative phenomenological analysis (IPA). Results: Five themes emerged from the IPA analysis: Bodily symptoms; relationships; building new identities; the future; and lessons learned. All the participating veterans gained a greater insight into and mastering of their condition, achieved better control of their lives, and developed tools to handle life situations more appropriately and to build a new identity. This improved their ability to participate in social activities and employment. Conclusion: The results should be considered in the future treatment of veterans with PTSD.

## 1. Introduction

Historically, nature has been used as part of a healing process for soldiers who have been mentally wounded in World War l. The first systematic use of nature in the shape of horticultural activities was developed during World War I [1,2] for soldiers suffering from shell-shock (later referred to as post-traumatic stress disorder), a prevalent condition in soldiers returning from the battlefield. These horticultural activities were first introduced as a voluntary treatment option, but gradually they became more professionalised, and today they are integrated into the treatment of occupational injuries [3,4]. Even though many programmes exist today, containing different kinds of nature elements, such as the wilderness, horticulture, and ecotherapy, scientific studies in this area remain sparse. To gain more knowledge, this study examines the effects of nature-based therapy (NBT) on a group of Danish veterans and shows that this form of treatment may be useful as part of the treatment offered to veterans with post-traumatic stress disorder (PTSD).

### 1.1. PTSD Diagnosis and Prevalence among Veterans

According to the *Diagnostic and Statistical Manual of Mental Disorders* (DSM-V) [5], a person diagnosed with PTSD must have been exposed to a traumatic event personally, or have witnessed a threat of death or serious injury, and subsequently experiences symptoms such as: Re-experiencing the trauma, for example, in flashbacks and nightmares; avoidance of places or activities that trigger memories of the traumatic event; arousal and reactivity associated with negative thoughts; aggressive behaviour that occurs with little or no provocation; and concentration problems.

Exposure to traumatic experiences during military service that leads to developing PTSD is the reality for many soldiers around the world, and the symptoms of the condition may cause extensive changes in the lives of veterans after homecoming. According to figures from the U.S. Department of Veteran Affairs, 15.8 per cent of veterans reported PTSD symptoms after having served in Iraq and Afghanistan [6]. An Australian study found that 8.3 per cent of veterans reported symptoms comparable with PTSD [7], while studies of British military personnel showed a lower prevalence, varying between 2.5 per cent and 6 per cent [8]. According to Richardson et al. [9], this large variability in prevalence may be explained by differences in the sampling strategies and diagnostic criteria used in the studies. The DSM-III-R criteria did not require ‘clinically significant impairment’ for the diagnosis of PTSD to be made. However, this criterion was introduced in the DSM-IV, which ultimately decreased the prevalence rate of PTSD. Richardson et al. [9] also suggested that the duration and intensity of combat exposure during deployment may influence the prevalence of PTSD. Furthermore, the prevalence of veterans reporting PTSD symptoms after homecoming is expected to increase [10,11,12]. The reasons for this increase have not been fully documented, but a greater awareness of PTSD and more focus on breaking down the stigma surrounding mental health issues may be underlying causes [13].

In accordance with DSM-V [5], symptoms of PTSD must be present within six months after the traumatic experience. However, there is growing awareness of the condition called ’delayed-onset PTSD’, where the symptoms appear later than six months after the trauma. A review by Andrews et al. [14] found that delayed-onset PTSD is 33 per cent more common in veterans compared with in the civilian population, and that the delayed onset of symptoms is likely to occur within 18 months after leaving the military. A recent study from Australia found the prevalence of delayed-onset PTSD among Australians veterans who served in Iraq or Afghanistan to be as high as 16.5 per cent [15]. In the UK, the prevalence of delayed-onset PTSD [16] in veterans increased from 3.6 per cent in 2007 to 6.9 per cent in 2009. The same tendency is found in Denmark, where 2.4 per cent of veterans who served in Afghanistan were identified with symptoms compatible with PTSD immediately after homecoming. This number had increased to 9.7% three years after homecoming [12]. Therefore, it can be assumed that in the future, the numbers of veterans suffering from PTSD will increase over time.

### 1.2. How Living with PTSD Affects Veterans’ Lives

PTSD symptoms may affect the lives of the veterans themselves and their families at a physical, emotional, and relational level. Research also suggests that there is a relationship between PTSD and general health. Hoge et al. [17] found that, in war veterans, the PTSD diagnosis was significantly associated with lower ratings of general health and more missed work days, as well as a higher incidence of physical symptoms and pain [18,19]. Several studies [20,21] have also found alcohol and drug abuse, depression, and anxiety disorders to be prevalent comorbidities to PTSD. Milliken et al. [22] stated that 12 to 15 per cent of veterans reported problematic alcohol use three to six months after returning from combat.

In addition to these health-related problems, social and family problems are also related to living with the PTSD diagnosis [9,23]. A review from 2004 [24] found that the individual’s traumatic stress had a negative effect on family members. In a recent study, Vogt et al. [25] found that PTSD was associated with poorer work and family functioning and satisfaction, and that the most consistent negative effects were on intimate relationships. Moreover, numbing, high arousal levels, and anger have been found to be associated with secondary traumatization and troubled families [26,27], and veterans suffering from PTSD have been shown to be four times more likely to contemplate suicide than non-PTSD veterans [24].

In addition to causing significant problems for the individual, PTSD involves massive economic challenges for society. The RAND report from 2008 [27] estimated that PTSD, major depression, and traumatic brain injury two years after deployment can cost up to $25,000 per case in the US. Providing evidence-based treatment to everyone could reduce these costs by up to 27 per cent [28].

### 1.3. Conventional Treatments for Veterans with PTSD

Medication and psychotherapy, such as behavioural and cognitive therapy, are conventional and common treatments for veterans diagnosed with PTSD. For example, Puetz et al. [29] found that SSRIs (selective serotonin reuptake inhibitors) and tricyclic antidepressants had an impact on anxiety and depressive symptoms in combat veterans. Sleep disturbances, which are common symptom of PTSD, have also been found to be alleviated by pharmacology treatment [30]. Even though many veterans feel that medication helps reduce their PTSD symptoms, the negative side effects they experience are substantial, and this leads to many of them giving up on medication [31].

Psychotherapy is a common alternative to pharmacotherapy. In a guideline from 2017 [32], the US Department of Veteran Affairs recommends: “Individual, manualized trauma-focused psychotherapy over other pharmacologic and non-pharmacologic interventions for the primary treatment of PTSD. Cognitive content and processes are seen as central to theories of understanding PTSD” [32]. In line with this, numerous studies have shown that trauma-focused cognitive behavioural therapies (CBT) such as cognitive processing therapy (CPT), prolonged exposure therapy (PE), and other cognitive therapies have a positive effect on PTSD symptoms by reducing destructive or disturbing behavioural patterns [7,33]. Moreover, a meta-analytical review [34] has shown that half of the combat veterans treated with a variety of psychotherapeutic interventions experienced an improvement in their symptoms compared with a control group.

In addition, the impact of non-trauma focused treatment has been evaluated. Present-centered therapy is an example of a non-trauma focused treatment that has been found to have a positive impact on PTSD in several studies [33,35,36,37,38]. Moreover, EMDR (eye movement desensitization and reprocessing) has been shown to have a positive effect in some studies [39,40], and mindfulness has also been shown to be effective with regard to PTSD symptoms [41,42,43,44] in a number of recent studies.

### 1.4. How NBT Can Be Seen as Part of a Treatment for Veterans with PTSD

Despite having a long history, NBT has, as mentioned above, only been sparsely investigated as a treatment option for war veterans. NBT may have some advantages relative to traditionally employed therapies that have high dropout rates [36,45,46] and that are sometimes experienced by veterans as reinforcing their symptoms [47]. For veterans, seeking help for mental problems is often related to perceived stigma [48,49,50]. Receiving therapy in nature might be an easier threshold for veterans to cross than engaging in more traditional therapy. In addition, PTSD may lead to cognitive impairment with concentration problems which might hamper the effect of the conventional therapies, and in some of the individuals, the cognitive focus could reactivate the trauma. Therefore, a therapy where the focus is not directly on thinking and reflection might be more acceptable [28,39,51]. Consequently, it would seem relevant to follow the historical thread from WWl with introduction of the first horticultural activities for war victims and offer the treatment to veterans with PTSD, along with an investigation of the participants’ experiences of the intervention. NBT as a treatment for mental illness builds on a solid evidence [52,53,54,55]. NBT has been shown to be effective in the treatment of depression [56,57,58,59] and with regards to individuals with PTSD, depression is a frequent comorbidity [60,61].

### 1.5. Nature-Based Therapy (NBT)

NBT is often used as an umbrella term for therapy based on experiences and activities in a natural setting that are specifically identified or designed to support the treatment process. Different nature-based therapeutic activities using horticultural activities are derived from occupational therapy [62,63]. In the UK and the US, NBT developed into a discipline of its own, based on the successful rehabilitation of war veterans. The background for using nature as an important part of the intervention is grounded in attention restoration theory (ART), which was developed by Kaplan and Kaplan [64].

ART suggests that humans have two types of attention: Directed attention and soft fascination. Directed attention is used when the individual has to concentrate on important matters. This process requires effort and can cause mental fatigue over time. In contrast, soft fascination captures the individual’s attention effortlessly. Soft fascination is at play when we, for example, are looking at clouds moving across the sky, and this kind of effortless brain function has been shown to lead to recovery from stress-related illnesses [65]. In relation to the treatment of war veterans suffering from PTSD, other researchers have pointed to ART as a valuable theory with which to understand the benefits for veterans of being in nature environments, e.g., [40,66,67]. Hawkins et al. [66] explained that nature, for many veterans, is experienced as emotionally calming. He refers to Kaplan’s [68] descriptions of nature as having a buffering effect that reduces the adverse effects of stressors (high arousal and rapid response to sudden sensory input), in other words, nature is seen as a meaningful place. This also holds true for many veterans, who often feel related to nature because of the many hours of training they have spent there, and they experience a sense of freedom when they are in nature [69,70].

Therefore, the aim of this study is to explore how war veterans experience living with PTSD on a daily basis during and after a ten-week nature-based therapy in a therapy forest garden.

## 2. Materials and Methods

This is a qualitative study. Data material consisted of semi-structured individual interviews and one focus group interview with eight Danish war veterans diagnosed with PTSD. This paper is part of a larger study that explores how a nature-based intervention in a therapy garden was experienced by veterans with PTSD, with a focus on the daily experiences of living with PTSD.

### 2.1. Setting

**Figure 1:**
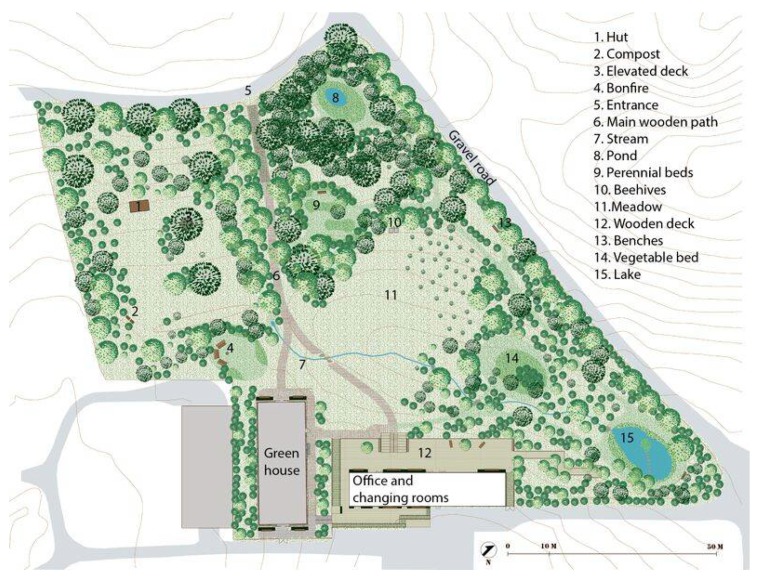
Figure one is a map of the therapy garden, Nacadia.

**Figure 2:**
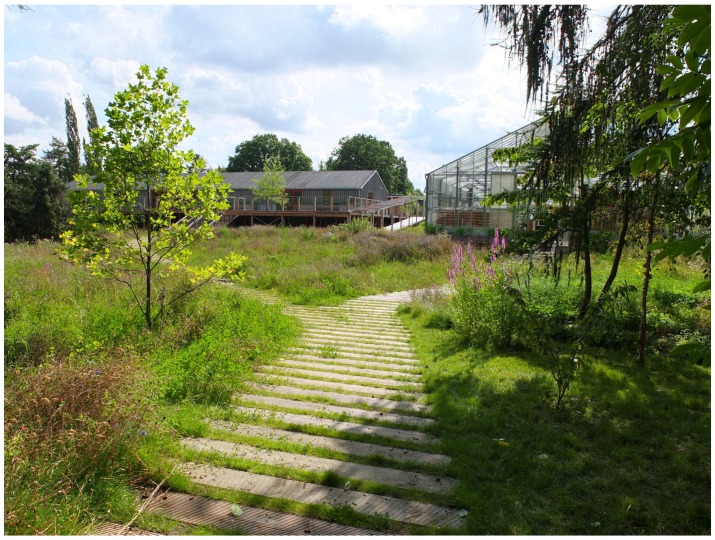
The picture shows a view from the therapy garden, Nacadia.

The NBT took place in the University of Copenhagen’s therapy forest garden, Nacadia, located in the arboretum. The garden is designed in accordance with a model for evidence-based health design [71], with activities contributing to NBT. Figure 1 gives an overview of the features in Nacadia. The design provides possibilities for nature-based activities (NBA) all year round, and its design and treatment programme are closely related. Figure 2 shows the therapy garden seen from the main wooden path just before crossing the stream.

### 2.2. The Therapists

Two horticultural therapists with psychological backgrounds were primarily responsible for the daily programme. The arboretum supervisor initiated tasks of a more physical character. A psychiatrist and a medical doctor conducted the weekly psychotherapeutic conversations in the garden area.

### 2.3. Nature-Based Therapy

NBT is based on the following elements: Horticultural and body-awareness activities supplemented by individual therapeutic talks. The value of combining these elements is described by Corazon et al. [71]. The mindfulness or awareness activities aim to reduce the physiological effects of stress exposure and to re-establish healthy flexibility to the nervous system. Working with the psychological trauma when appropriate and implementing nature and mindfulness-based tools for coping with PTSD are considered to enhance the ability to handle challenging life situations.

The length of the NBT was ten weeks, with three hours of therapy three times a week. The duration of the weekly individual therapeutic conversations was one hour. It was part of a therapeutic approach to encourage the veterans to be aware of and respect their bodily sensations, and use this knowledge to choose activities matching their mental and bodily capacity. The intention was to build up the veterans’ self-confidence and experience of success in the activities.

The intervention had a daily structure, the purpose of which was to create a recognizable and safe frame for the participants. The day started with a walk through the arboretum forest to the therapy garden. Upon arrival, a gathering around the fireplace was the first item of the day. Body awareness is considered as potentially beneficial for health [72]. Body-awareness exercises like ‘body-scan’, mindful awareness of breathing, and sounds and thoughts in the nature-setting were used. The aim was to reduce the high arousal level, and bring calmness into the nervous system. This was followed by horticultural activities and private time.

The horticultural activities involved a physical element, e.g., wood splitting and planting of trees that could benefit their needs for physical challenges [70], as well as more relaxing activities conducted alone, with staff members, or in the group.

### 2.4. Participants

Potential participants were recruited through advertisements in newspapers and on websites for soldiers and veterans. Those who had served abroad and were diagnosed with PTSD could apply for participation and could visit the therapy garden. The next step was a meeting with members of the staff. Eight male veterans, aged 26–47 years, were included in the project. Veterans with psychotic conditions were excluded.

With regard to the veterans included in the study, the time since they had first received their PTSD diagnosis ranged between nineteen months and two years, while the time until onset of symptoms after service ranged from a few months to more than eighteen years. The veterans had served in war zones for one to four periods (of six months) in the Balkans, Iraq, and Afghanistan. Seven of the included veterans used medicine for mental PTSD symptoms, such as anxiety, anger, and sleeplessness. Drugs (not prescribed by physicians) formed part of the self-medication for five of the participants. Three participants from the group were working part-time at the military barracks. The remaining five were on sick leave for a period between two to twelve years, and received unemployment benefits.

Dropouts: After three weeks, one of the participants developed psychotic symptoms and was therefore excluded from the study. One participant started an education two weeks before the NBT ended.

### 2.5. Data Collection and Analysis

Before the intervention, the first author conducted initial talks (pilot interviews) with veterans with PTSD (non-applicants). This resulted in a pre-understanding of the experiences and the physical and mental wounds that can arise during active service. This recognition was used to develop the interview guide and the appropriate approach during the data collection. Interview guides were created, with the aim to explore how veterans live with PTSD on a daily basis and their experiences of how NBT affected their lives.

Four individual, semi-structured, open-ended interviews were carried out with each participant. The first interview was conducted before the intervention, the second after five weeks, the third after ten weeks (at the end of intervention), and the last interview was conducted one year after the intervention. There were separate interview guides for all four interviews. The first interview focused on the veterans’ previous relation to nature, their experiences with activities in nature, and how they perceived having PTSD, including bodily changes and sensations, their coping tools, and experiences when not being able to alleviate symptoms. The focus of interview two was on the veterans’ experiences with the therapy garden and changes in symptoms and function during the therapy, and activities of special importance. In the third interview, the questions were once again about changes in wellbeing, but there was also a focus on cooperation and fellowship with the group and future relations to nature. The last interview, one year after the termination of therapy, was about the veterans’ wellbeing, their continued relation to nature, and their evaluation of the effect of the therapy on their daily life. The interviews lasted between 46 and 94 min. In addition, one focus-group interview with all participants was conducted after ten weeks of treatment.

During the interviews, it was considered important to generate an atmosphere in which the participants felt physically and emotionally safe. This was achieved by, for example, letting the participants decide where the first interview should be conducted. Four of the participants chose their homes due to experiencing anxiety when venturing outside the home. Two interviews were held in the military barracks, and two at the interviewer’s office.

The second interview was conducted in the therapy garden while walking on paths chosen by the participants. The third interview was conducted in the therapy garden near the bonfire area, while the fourth interview took place in the participants’ homes or at the interviewer’s office.

Due to dropouts, seven veterans participated in the first three interviews. By the end of the fourth interview one year later, five veterans participated.

Notes about the interview situation or the participants’ physical reactions were taken during the interviews, in order to include the circumstances around the statements and body language that might reveal knowledge or improve the interviewer’s opportunities to ask clarifying questions [73,74]. The notes were added to the transcriptions. All interviews were conducted, digitally recorded, and transcribed verbatim by the first author.

### 2.6. Data Analysis

Interpretative phenomenological analysis (IPA) was used to analyse the data. IPA has its roots in phenomenology, and the method aims to explore in detail the participants’ life-world and their personal experiences and perception [75,76]. The method involves a dynamic process with the researcher playing an active role and, thus, acknowledges the influence of the researcher’s personal conceptions and the interpretative activity involved [77]. The method is used increasingly within the psychological field, as well as in social research [78,79]. Data were analysed following the stepwise procedure of the IPA method: Repeated readings of each interview to gain an overall impression, notes on passages of special interest, followed by identification of initial themes that capture the essence of the interview [75]. This procedure was repeated with all interviews, and initial themes were identified. Following this, themes were clustered, common themes were identified across the interviews, and, finally, main themes were identified. This formed the basis for the narrative writing up. The first author conducted the first steps of the analysis process, including the first clustering of themes and identification of main themes, together with the third author, who is a qualitative researcher with experience in the IPA-method. The analysis process was discussed among all authors, and they all participated in the process of identifying the five main themes. Divergent views were discussed until agreement was reached.

## 3. Results

The analysis resulted in five superordinate themes: When the body speaks; relationships, imperative and unbearable; building a self; the future, dreams, fears and hopes; and lessons learned. These themes will be elaborated below.

### 3.1. When the Body Speaks

All the veterans reported that they had experienced bodily sensations before being diagnosed with PTSD. Some weeks or months after returning home, they noticed changes in their bodily reactions. They complained of headaches, memory impairment, sleep disturbances, and increased sensitivity to sounds that triggered memories of the war, for example the sound of a helicopter or an accelerating motor vehicle. They said that, at first, the bodily symptoms were confusing and they did not relate them to a possible PTSD diagnosis. Some described it as an experience of being out of touch with their bodies, and of feeling a need to escape physically by running away or mentally by sleeping:

“I was like a volcano; I felt the pressure inside and out. Suddenly it says BOOM. Sometimes I felt like I had to run as fast as I could to get away from myself.”

Most of the veterans said that they had sought help at a hospital for their symptoms, because they experienced the symptoms as being physical and in some cases even life-threatening, as if they were having a heart attack.

”First there’s a pressure on my chest, like a child pressing, then it tightens like a band round my trunk, and in the end, it’s like an elephant planting a foot on my chest, pressing so hard I can’t breathe.”

The medical examinations did not lead to a physical explanation for their symptoms. Some began to consider whether there was a connection between their physical problems and their mental state and contacted the military health service. Others said that they continued to search for a physical diagnosis. A few described it as a struggle with large personal costs; it was no longer a question of getting a diagnosis, but of being respected as a person.

”I have struggled tremendously the last year to make them aware of my problems […] It has been the most stressful thing in my whole life. I just hope that I will be treated as I deserve.”

During the last five weeks of the intervention, all the veterans described changes in their bodily symptoms. These changes occurred at a different pace and did not always lead to a complete absence of symptoms. However, the veterans talked about how even a small change led to the realisation of how their symptoms had influenced their daily lives. In the third interview (toward the end of the intervention), one of the veterans mentioned that he had been able to sleep through the night for the first time in 18 years. Others talked about better quality of sleep, or about managing to let go of the anxiety connected with falling asleep and the nightmares the sleep might bring:

“My dreams… I have positive dreams now. I still have some of those dreams that are hard to handle, but I am more distanced from them. Like standing outside and looking down on it. It does not affect me in the same way. More relaxed, you can say.”

The veterans said that the feelings of turmoil and of being in a state of constantly heightened alertness was reduced during the nature-based intervention. Most of the veterans experienced a peaceful feeling in their bodies during the nature-based activities. One said that it was as if breathing had become easier and this had led to reduced tension in his muscles. Another mentioned that this relaxed condition was a big motivator for doing some of the activities that he liked less:

“All that yoga and stuff, I do it because I am looking forward to lying down and listening [] … I love to be in that trance-like state…I’m almost counting down.”

In the second interview (halfway through the intervention), all the veterans stressed that the breathing techniques had become a useful tool for coping with stressful situations, such as sitting close to other people on a bus or going shopping in a crowded supermarket. They also told that they used to be very sensitive to other people’s emotions, but that this had changed during the intervention. One of the veterans described this change in the third interview:

“I feel like the body is relaxing more. …normally I feel like a radar for people’s anger, even though it doesn’t have anything to do with me. If I go down the street and see someone who is angry, my system is up high [..]. Now I can tell myself it has nothing to do with me.”

This change in behavioural patterns made it possible for the veterans to participate in social activities.

### 3.2. Relationships, Imperative and Unbearable

Two types of relationships were described as being affected by the veterans’ condition: Relationships with soldier comrades, and relationships with family and friends. The veterans described in detail the close relationships they had with comrades that had developed during service. They had felt a responsibility toward each other in their everyday lives, which had given them a sense of safety in spite of living with the constant threat of attack. The veterans described this fellowship with other soldiers in the first interview:

“Your comrades are the ones you would die for…and the ones who help you handle your problems.”

When a comrade became wounded or died, the veterans experienced grief and guilt because they were alive. One of the veterans expressed it as follows:

“I feel guilty about being alive, and that I did not bring my comrade back home with me, even though I promised his mum I would.”

This veteran said that he had taken on a personal responsibility and that he blamed himself for his friend’s death. The fact that he had promised his friend’s mother that he would bring his friend home made it even worse. Two of the participants were sent home for treatment before they had finished their posting and recounted how they felt ashamed about leaving their comrades behind.

Being on sick leave stimulated thoughts about what others might think of them, and made them feel that they were seen as cheats who had invented the symptoms themselves. Some felt it as if other people were talking badly about them behind their backs:

“That guy, is he really sick?”

In the first interview, most of the veterans described a feeling of loneliness after coming home, and some felt an urge to isolate themselves from their relatives and friends. They said that during service, they considered their comrades to be replacements for their families. When they first returned home, their families could not replace this relationship. The veterans described their relationships with their families as difficult and often contradictory; on the one hand, their families were the most important thing in the world to them, while on the other hand, they could not handle being together with them. All the veterans thought that they had changed and were different from how they had been before service, and they found it difficult to meet their families’ needs for affection. One veteran, when talking about his girlfriend, who used to call him or send him messages every day, said the following:

“I haven’t got the room inside me to be there for her every day… I can’t… for me feelings are just not like before.”

In the first interview, the veterans also emphasised how they had changed after they had served; they were now in a constant state of alertness and unable to refrain from observing people’s behaviour when in a crowd. They said that they always felt the need to check for snipers and hide under tables when hearing the sound of airplanes. This behaviour frightened other people and was seen as being extreme. They felt that it had a significant effect on their ability to take part in social arrangements and it also led to unintended suspicion from their families. One veteran related the following episode about his wife coming home from work:

“I look at her…her hands, if she is carrying something, keys or… or if something looks different. If her hand is closed, I have to follow her movements until I have seen what she has got there.”

All the veterans described how both physical contact and being able to sense affection were hard after coming home. One of them was unable to pick up his little son:

“It has been terrible. Especially for my son. He has his needs… [interviewer: And you can`t give this to him?] “No, I can’t take it [touching and skin-contact].”

They also said that they experienced difficulties sharing feelings and talking about how they felt, which made their emotional communication with their relatives difficult:

“It’s hard for me to express how I’m feeling and maybe also hard for them to know what to say to me [...] Not even my family knows much about how I’m doing. It stays inside me, and it’s very very hard to open up and talk about how I’m feeling and thinking.”

In the interviews conducted midway through the intervention, the veterans emphasised some changes that had occurred in the way they perceived relationships. They all experienced a positive feeling of being part of the group of veterans during the NBT. Being surrounded by people with the same background, similar experiences, and challenges was considered more comforting than being with civilians.

“If I had started in another group, where people had PTSD but for civil reasons, I don’t think it would have been the same… we have this mutual understanding [...] There are things we don’t need to say… we know what it’s like to serve abroad and be in a state of constant alertness.”

This understanding resulted in an atmosphere in the group in which they felt free to put memories and reflections into words. They expressed the importance of being in an ambience of understanding where no explanations were needed.

In the same round of interviews as above, several of the veterans highlighted how the term ‘acceptance’ had become a cornerstone in their thinking about life. Gradually, the veterans expressed acceptance of what life might bring instead of fighting it. In addition, during the NBT, the veterans described different strategies they had developed to cope with stressful situations and quarrels with their families.

### 3.3. The Future, Dreams, Fears, and Hopes

In the first interview, all the veterans related a history of long-term treatment with medication and/or psychotherapy. Some of the treatments had provided small improvements, but had also shattered hopes and dreams when the improvements did not last or even failed to materialise. Even so, in the first interview, all the participants expressed positive expectations to the intervention. However, their expectations differed and reflected their present life situation. Some felt the need for specific tools to help them to regain control:

“I need an instrument to control the turmoil and nerves in my body because I hate it. I hate having it […] it’s a heavy burden.”

Several of the veterans’ statements reflected their hopes and fears regarding their PTSD symptoms. Being disappointed could affect their mood and reinforce anxiety. For the veterans, the alternative to not getting better was frightening. One veteran with a background as a social worker said in the second interview:

“You get anxious of this change [of oneself]. And where does it lead to? You see the weirdo on the street, and suddenly you imagine that’s the way you’re going.”

Another participant was unable to see a future for himself. He expressed it like this in the second interview, giving an impression of the deep worry that the veterans face:

“Future? It’s almost like it’s just some letters on a piece of paper that I see. A mathematical formula on a white wall…”

However, at the end of the intervention, the veterans’ thoughts about the future had changed. Anxiety was considered less dominant. Instead, the veterans accepted the slow pace of the improvements they experienced, and time became an important factor in their healing processes. They expressed an awareness that they should not put pressure on themselves.

“Before, I was so preoccupied with saying, in six months I will have got so far with my disease. But I’ve dropped everything to do with time horizons and that sort of thing. Things must come as they are, the more I hurry, the more I stress myself, and the less energy I have to handle things.”

This expresses a growing insight into the condition among the veterans, and it acknowledges their own role in a recovery process. During the intervention and the year after the NBT, some of the veterans even experienced being able to handle their problems and imagine a future. A veteran expressed it like this in the final interview (one year after the intervention):

“Now I manage to do some of those things that I had completely avoided before […] Yes I have a dream…being more independent [financially] through having a job. Sometimes I really wonder if I am that damaged that it’s a utopia for me…but, then I think, if I really could make it, I would create a life together with my son.”

### 3.4. Identity—Construction of a Self

The veterans described their identities as soldiers as strong and important parts of themselves in the first interview. Coming home, feeling different, and receiving a PTSD diagnosis had a huge impact on their lives. They said that being constrained and not being able to do the same things as before had resulted in their view of themselves as having changed, and this forced them to work on constructing another identity.

Almost all of the participants were young and had recently graduated when they were sent to war areas. They experienced how life as a soldier in a war zone often differed from their expectations, for example, that it was often difficult to distinguish enemies from civilians. The military rank system caused contrasting feelings: Some felt strengthened by rising up the ranks, while others felt the responsibility as a burden. There were also times when it was difficult to make situations fit into the rank system. One of the participants described a situation where he was accompanying a critically injured comrade into the helicopter to say goodbye. He felt this existential point between life and death where emotions exceeded the military rank within the group.

“Then my boss comes up to me and grabs me. I remember, I tried to get away, but then he gave me a hug. And then it starts! A tiny little spark… And then the priest comes over, and he’s a fucking good priest. He’s like a dad. He always blesses us when we go on patrol, and it might look silly on television, but it’s bloody important when you’re in a place like that.”

All the veterans expressed in the first and second interviews that fellowship, the uniforms, the weapons, and the common language of the military were important factors which bonded them together and gave them a common identity. One of them expressed (15 years after leaving the military) how he still felt strongly connected to his weapon:

“Well it’s a bit strange, but when I was in the military, I was walking in the night, it was much more comfortable if I had my rifle, although it was secured and only fired blanks…it was still my weapon, and it gave me some calmness and protection […] and even when I walk around as a civilian, I miss it, walking at night.”

Some veterans described that after returning home and experiencing the changes as a result of PTSD, their self-perception gradually turned into a feeling of alienation. One felt like he was just an observer of his own life instead of actually living it:

“I’m a stranger in the world, but also to my own body, and the only thing that ties me to the world is a sewing thread.”

The NBT provided an insight into the participants’ self-perception. During the first interview, one veteran described how he was no longer *“a master in his own house.”* This can be interpreted as PTSD had taken over the control of his life. However, one year later, in the last interview, when asked the same question, he answered:

“I’ve found out that I don’t even have the foundations of the house…you build up a little, and find out that it must be in another way because it doesn’t work for me that way.”

The participants said that when they were no longer able to serve as a soldier, finding a new occupation became an important task in constructing a new identity and being a respected member of society. When talking about his new job, one veteran said (interviewed one year after NBT):

“It’s a place where I feel respected, and they talk to me as a human being and not just a worker, so I want to give more, and I can push myself more […] I’ve got my professional pride back.”

Being able to handle one’s own situation is a huge step in gaining control over life in spite of having PTSD.

### 3.5. Lessons Learned, Reflections

During the therapy, the veterans experienced changes in their way of thinking and handling life situations presented in the four above themes. ‘Lessons learned’ can be seen as the veterans’ own reflections on the whole process, from starting the NBT, to one year after it had ended.

The veterans expressed how the NBT helped them take more initiatives (for example, they became gradually more socialized) and find solutions (they could now use different tools) to the restrictions in their daily lives with PTSD.

“So I try to do different things…there’s a dog’s playground next to my place, and for a period, I sat on a bench nearby. Partly not to be too close, but on the other hand, I wanted to be so close that I got some human contact without being in focus.”

One veteran reflected on how a better understanding and acceptance of himself could be a stepping stone to gaining more control of his life. However, it could also imply extra work:

“One might think about things in a totally different way now, and it [the PTSD symptoms] makes you rise to the challenge, but at the same time, you become more handicapped…or what you might say.”

He used the word “handicapped”, which is usually associated with a physical disability. However, the term expressed his feeling of being unable to function on equal terms with others.

Accepting one’s needs and capacities was part of the NBT. It was a challenge for some of the participants to implement acceptance in their daily lives, as this was considered to be in opposition to structure and order as an expression of being in control. One veteran described how he had felt that untidiness and things being out of place was unacceptable before he took part in the NBT. However, now this was seen as a positive thing:

”I’ve found out that everything does not have to be exactly in order all the time, right? Before, it meant a great deal to me that everything was in the right place. I think it’s because I found out that my head can’t deal with it.”

It takes time to implement some of the content from the NBT. During the third interview, one veteran talked about a frustrating episode in the garden: The horticultural therapist had asked all the veterans to hold hands in a circle and this was something he felt very uncomfortable doing. After a while, he spoke up and the therapist praised him for doing so:

“I was not the only one who felt that way,—but we are soldiers and follow orders [..]”

During the final interview, he mentioned the same episode again and said:

”I have changed now because I dare to say ‘no’, and it will be easier for me in my future life.”

## 4. Discussion

First, a brief summary of the findings for each superordinate theme is presented. Then the results are discussed and related to the literature.

In general, the veterans experienced changes in their lives with PTSD from baseline to one year after the intervention. In the first interview, they described their bodily experiences from having PTSD as pain, sleep disturbances, high arousal, and flashbacks. For some, the changes occurred in the intervention period, while others described these changes in the last interview one year after the intervention. Not only had the symptoms decreased, the veterans also expressed a feeling of control in relation to distress.

The veterans described their relationships with their comrades during service as strong, and they felt guilty about leaving before completing their service or when comrades were killed. Being in a group of comrades that shared their experiences during the intervention helped them deal with the situation. Their relationships with family members were described as complex; the veterans wanted close relationships, but at the same time they were not always able to endure them. In the last interview, more veterans expressed how being in nature made them feel more calm, and how it was now less difficult to be with family and friends and to talk about their feelings.

All the veterans expressed a fear that the condition might worsen due to earlier experiences of highs and lows in relation to treatment. In the beginning of the intervention, the veterans expressed it as almost impossible to imagine the future. This changed during the intervention period. Through NBT, they seemed to gain more control over their lives, and it brought them to a state in which they dared to imagine a positive future.

The process of learning to accept the loss of their strong identity as a soldier and the creation of a new identity was described as demanding. For some veterans, being able to manage a job gave a feeling of being respected. During the intervention, the veterans improved their ability to find new solutions and strategies for handling challenges in their life in more appropriate ways.

When comparing the participants’ bodily reactions, they were in line with frequently reported literature [5,18,80,81]. PTSD symptoms can be understood from a biological, physiological, or sociological perspective. Bosco et al. [82] reviewed three models for understanding PTSD and chronic pain. They mentioned how different models, the fear-avoidance model, the mutual maintenance model, and the shared vulnerability model, might contribute to the different aspects of understanding pain in this group. There is now a consensus that this type of pain related to PTSD does not have an external cause [83]. Ruden [84] found that the pain was often associated with somatosensory changes and comorbidity with mental diseases. He suggested that during the traumatic event an encoding occurred, where a sense of powerlessness and inability to take responsive action could lead to pain as an outcome of the event. He explained that emotions produced in a fearful situation might cause delayed pain after the event, activated by a subconscious stimulus from the memory of which the person is not necessarily aware. The NBT seemed to change the veterans’ response to such situations and their reactions to their symptoms. The mindfulness exercises, where breathing exercises were used to maintain focus and bodily control [85], are believed to have had this impact. The stress-reducing effect of the intervention seems to have been useful. The NBT therapy garden was specifically designed to support people suffering from stress-related diseases [86,87].

The NBT offered the veterans an opportunity to join a group of fellow veterans. The veterans described how this fulfilled two of their needs: To be in a group representing the military culture with which they had lost connection, and to meet with people with similar experiences and PTSD symptoms.

The importance of relationships with comrades during service has also been identified in other studies [88,89], including studies of Danish soldiers [90] in which comrades were referred to as the ‘military family’. The special culture that prevails in the military and the impact on young soldiers is well described in the literature [91,92]. In this culture, the individual is dedicated to the country and his fellow soldiers with binding commitment to his unit. The concept of honor is important and fundamental for the function of the military [93]. However, this also implies that seeking help could be seen as a sign of weakness, and it might even be seen as dangerous to have people in need of psychological help on a combat team [88,94]. The veterans in the present study expressed the feeling of failure and guilt when comrades died, and inadequacy due to not being able to finish their time of service. Having mental instead of visual physical wounds was considered problematic; one veteran even said that he felt that others thought he was a cheat.

All the veterans said that their families were very important parts of their lives, but at the same time the relationships were also a source of problems. During and after the NBT, the veterans handled their relationships with their families differently. Emotional numbing and the feeling of dissociation from their families were described as reasons for the complicated relationships. While numbness may be a useful emotion during war, it complicated the veterans’ family relationships. Emotional numbness is listed as one of the PTSD symptoms that affects both the veterans and their families [5,26,94]. An important part of NBT is experiencing nature through the senses. Actions such as touching the bark of a tree, putting your hand in the stream and feeling the cold water, or listening to the birds activates positive memories and emotions. Establishing a relationship to nature can be a step toward establishing relationships to other human beings [95].

All the veterans who participated in the NBT wanted their lives to be less affected by PTSD. They asked for tools to help them reduce bodily stress and understand their bodily signals. This could be interpreted as an instrumental way of viewing the body as a ‘thing’ one must gain control over. This view may have its roots in the military system with discipline and bodily control [92,96], which has also been described by Foucault in his concept of “the docile bodies” [97]. However, through being in nature and doing therapeutic activities in nature, the participants learned some very concrete tools, such as breathing techniques, that facilitated a feeling of bodily control. Moreover, they also experienced a more spiritual awakening, for example, that seeking out nature can lead to an inner calmness. In addition, working with nature activities, such as producing bird boxes and hanging them on the trees in the garden, chopping firewood, or sowing seeds, was considered meaningful.

The design of the study made it possible to follow the effect of the NBT through the veterans’ experiences. The theme “lessons learned” addressed the veterans’ reflections regarding the effect. They mentioned acting more appropriately in difficult situations and using tools from the NBT as some of the positive effects of the therapy. NBT helped the veterans to develop dynamic strategies, which had a positive effect on their relationships, identity, and dreams for the future. Working with mindfulness activities seemed to give a deeper insight into themselves and their resources. The veterans described how working with ‘acceptance’ as part of the mindfulness activities gave them the opportunity to develop and deal with a new identity; maybe different from who they were before, but no longer an inferior version. Mindfulness conducted in a nature environment may have been tolerated better by the veterans, because many of the sounds like birdsong and running water are well known to the brain, and because no sudden noises, for example honking horns and people shouting, occur.

The NBT took place in nature and was an active contributor, both directly by simply being in nature, and indirectly through the horticultural activities. Kaplan [64] describes how soft fascination, which is stimulated by natural environments, has a restorative influence on individuals and helps reduce stress-related symptoms. Particularly the heightened arousal level that is part of PTSD symptoms seemed to be reduced by being in and sensing nature.

In the NBT, the therapeutic acceptance of the veterans’ own experiences of their resources and support of their choice of activities (e.g., relaxing instead of being more active) built up the veterans’ self-confidence. The veterans described how NBT influenced their ability to take the initiative and seek new solutions in their lives, and strengthened their capacity to construct a new identity. We do not necessarily see NBT as a treatment that is either a ‘stand-alone’ treatment or an ‘add-on’ therapy. The condition of the individual participant must be taken into account when determining the most beneficial treatment. Some of the veterans in this group had suffered from PTSD for just a few years, whereas as others had suffered from it for more than 18 years. Our results indicate that veterans who have been included in many ‘vet programmes’ or who have had bad experiences with medical treatment or who have felt that cognitive therapy was too provoking for their condition experienced the best results. This indicates that it is a suitable therapy for vulnerable veterans.

The study has some limitations. The participants were recruited through advertisements in newspapers and social media and webpages for soldiers and veterans. Therefore, our sample may have been comprised by a group of especially motivated veterans or veterans with especially mild or severe symptoms. In addition, only male veterans responded. However, the veterans in our study differed with regard to age and time since the onset of PTSD. In addition, they all represented rather well-known PTSD symptoms. Therefore, we think that our results are transferable to other male veterans. We cannot know if the results also apply to female veterans. As the study is qualitative, the question of generalisability cannot be answered. Nevertheless, the participants in our study experienced an effect of the intervention and the effect was sustained after one year. This means that the intervention is experienced as effectual by some male veterans.

## 5. Conclusions

The veterans of this study seemed to be split between two worlds. Living with the PTSD meant that at the beginning of the intervention, their awareness was in the military world; they had been enculturated to the military world with its extreme situations and they had lost the competences required to navigate in the civilian world. Their loyalty was also to the military world and was directed towards their comrades. They were locked emotionally in the traumatic situations they had experienced and brought their war identity home with them, where it did not fit in. They had to go through therapy to return home and adapt to a new civilian identity. The NBT helped them take on this new identity through a process where they, together with comrades, could gradually allow themselves to let go of their military identity. Even though they could not just take on their former identity again, the ability to relax again that they achieved through the experiences in nature helped them on their way to finding a new identity.

After the ten-week NBT programme in a therapy forest garden, the veterans diagnosed with PTSD reported that their bodily symptoms were less burdensome, and that being in a group with people with a similar military background and culture had been beneficial. Moreover, they noticed an improved ability to take part in social activities with their families. They had learnt to be more accepting of their condition and the slow pace of recovery, including the need for developing new identities. This opened a door to imagining a future again with an ability to work and improved self-esteem. The veterans’ reflections on the NBT process at the follow-up revealed that tools from the therapy had enabled them to better handle potentially difficult situations and gain more control of their lives. This means that the results seem sustainable. In the last interview, all the veterans expressed that living with PTSD had become easier. Although the study is based on a small number of informants, the results seem promising and NBT should possibly be considered as a therapy complementary to the existing evidence-based therapies.
